# Implications of disease-modifying therapies for multiple sclerosis on immune cells and response to COVID-19 vaccination

**DOI:** 10.3389/fimmu.2024.1416464

**Published:** 2024-07-15

**Authors:** Valeria Orrù, Valentina Serra, Michele Marongiu, Sandra Lai, Valeria Lodde, Magdalena Zoledziewska, Maristella Steri, Annalisa Loizedda, Monia Lobina, Maria Grazia Piras, Francesca Virdis, Giuseppe Delogu, Maria Giuseppina Marini, Maura Mingoia, Matteo Floris, Marco Masala, M. Paola Castelli, Rafaela Mostallino, Jessica Frau, Lorena Lorefice, Gabriele Farina, Marzia Fronza, Daniele Carmagnini, Elisa Carta, Silvy Pilotto, Paola Chessa, Marcella Devoto, Paolo Castiglia, Paolo Solla, Roberto Ignazio Zarbo, Maria Laura Idda, Maristella Pitzalis, Eleonora Cocco, Edoardo Fiorillo, Francesco Cucca

**Affiliations:** ^1^ Institute for Genetic and Biomedical Research, National Research Council, Lanusei, Italy; ^2^ Department of Biomedical Sciences, University of Sassari, Sassari, Italy; ^3^ Institute for Genetic and Biomedical Research, National Research Council, Monserrato, Italy; ^4^ Department of Biomedical Sciences, University of Cagliari, Monserrato, Italy; ^5^ Regional Multiple Sclerosis Center, Azienda Sanitaria Locale (ASL) Cagliari, Cagliari, Italy; ^6^ Neurology Unit, Azienza Ospedaliera Universitaria (AOU) Sassari, Sassari, Italy; ^7^ Department of Medical Science and Public Health, University of Cagliari, Cagliari, Italy; ^8^ Department of Translational and Precision Medicine, Sapienza University, Rome, Italy; ^9^ Department of Medicine, Surgery and Pharmacy, University of Sassari, Sassari, Italy

**Keywords:** SARS-CoV-2, multiple sclerosis, disease-modifying therapy, immune response, immune-phenotyping

## Abstract

**Introduction:**

Disease-modifying therapies (DMTs) have been shown to improve disease outcomes in multiple sclerosis (MS) patients. They may also impair the immune response to vaccines, including the SARS-CoV-2 vaccine. However, available data on both the intrinsic immune effects of DMTs and their influence on cellular response to the SARS-CoV-2 vaccine are still incomplete.

**Methods:**

Here, we evaluated the immune cell effects of 3 DMTs on the response to mRNA SARS-CoV-2 vaccination by comparing MS patients treated with one specific therapy (fingolimod, dimethyl fumarate, or natalizumab) with both healthy controls and untreated patients. We profiled 23 B-cell traits, 57 T-cell traits, and 10 cytokines, both at basal level and after stimulation with a pool of SARS-CoV-2 spike peptides, in 79 MS patients, treated with DMTs or untreated, and 32 healthy controls. Measurements were made before vaccination and at three time points after immunization.

**Results and Discussion:**

MS patients treated with fingolimod showed the strongest immune cell dysregulation characterized by a reduction in all measured lymphocyte cell classes; the patients also had increased immune cell activation at baseline, accompanied by reduced specific immune cell response to the SARS-CoV-2 vaccine. Also, anti-spike specific B cells progressively increased over the three time points after vaccination, even when antibodies measured from the same samples instead showed a decline. Our findings demonstrate that repeated booster vaccinations in MS patients are crucial to overcoming the immune cell impairment caused by DMTs and achieving an immune response to the SARS-CoV-2 vaccine comparable to that of healthy controls.

## Introduction

Disease-modifying therapies used in MS patients aim to reduce the number of relapses and improve the course of the disease. However, their broader effects on the immune system, particularly on immune cells, are only partially understood and may influence immune response to pathogens and vaccines, including that against SARS-CoV-2.

Previous studies have mainly evaluated humoral response (1–4) or cytokine production after SARS-CoV-2 vaccination (5–8) and few focused on dissection of both the B and T cell compartments of the cellular immune response (9, 10). Some have studied the effects of only a specific treatment, such as anti-CD20 antibody drugs (11, 12), or only a specific time after vaccination (13–18). Apostolidis and colleagues (11) studied the vaccine response against SARS-CoV-2 in MS patients treated with ocrelizumab and rituximab (monoclonal antibodies directed against the B-cell surface antigen CD20). The consequent B cell depletion greatly reduced the humoral response and, more in general, the B-mediated response, without altering the T cell compartment. However, no more systematic study has been carried out for other MS treatments that alter the immune system. Indeed, in fingolimod-treated patients, booster vaccination was described to augment humoral response, but cell activation after SARS-CoV-2 vaccination was not well documented with a main focus on cytokine production (7, 8). Similarly, dimethyl fumarate and natalizumab therapies are known to dysregulate the immune response in treated patients, without precluding an effective seroconversion after vaccination (3, 4, 15), but only part of the B and T cell response was studied (5, 17, 19, 20).

To overcome these shortcomings, we have examined the effects of these therapies on the cellular (both B and T compartments), humoral, and cytokine response to SARS-CoV-2 vaccine over time after challenge.

We measured the cellular response and levels of 10 cytokines at baseline and after stimulation with a pool of spike peptides, before and at 3 times after SARS-CoV-2 vaccination, in patients stratified by therapies compared to untreated patients (UNTR), and healthy individuals (CT). The cellular response was also correlated with antibody production.

We studied the DMTs most used in our cohort of MS patients, namely fingolimod (FTY), dimethyl fumarate (DMF), and natalizumab (NAT), for their effects on response to SARS-CoV-2 vaccination. FTY belongs to a class of anti-inflammatory immunomodulators that inhibit the egress of T cells and B cells from the thymus and lymph nodes (21); DMF is the methyl ester of fumaric acid, which has anti-inflammatory properties and axonal regenerative function (22); and NAT is a monoclonal antibody against the cell adhesion molecule alpha-4 integrin, which inhibits the passage of inflammatory immune cells across the blood-brain barrier (23).

The approach used here can be considered as a model study of the immune response to external stimuli, asking in this case for any differences in the immune response in MS patients stratified for DMTs with different mechanisms of action.

## Methods

### Studied participants

We evaluated a subset of Sardinian MS patients and controls described in a previous work on humoral response after SARS-CoV-2 vaccination (9). Briefly, 79 MS patients were recruited in the MS clinical centers in Cagliari and Sassari in Sardinia (Italy). MS patients were diagnosed according to McDonald criteria. Thirty-two healthy individuals from the SardiNIA general population cohort (24) were recruited as a control group. Patients and controls received two deltoid injections, 21–28 days apart and a further dose six months after the second dose. Each injection contained 30ug/dose of BNT162b2 (Pfizer) or 50–100ug/dose of mRNA-1273 (Spikevax, Moderna). The main characteristics of MS patients and controls are described in **Table 1**.

**Table 1:** Summary features of the assessed individuals. Clinical and demographic characteristics of MS patients stratified by therapy (FTY, DMF, or NAT), left untreated (UNTR), and healthy controls (CT) that received SARS-CoV2 vaccination.

Spike B panel	Sample size				Age in yrs, median (min-max)	Disease duration in yrs, median (min-max)	EDSS median (min-max)	% females
	T0	T1	T2	T3				
**FTY**	11	18	17	24	45 (30-67)	22 (10-50)	2 (0-6.5)	80
**DMF**	19	19	17	23	48 (27-63)	18 (6-44)	2.5 (0-6)	74
**NAT**	15	12	18	20	43 (22-59)	12 (2-39)	1.5 (0-6)	80
**UNTR**	8	11	3	12	55 (36-79)	24 (1-60)	2 (0-9.5)	92
**CT**	7	28	7	32	51 (29-63)	na	na	79
B-T panel	Basal sample size				Stimulated sample size			
	T0	T1	T2	T3	T0	T1	T2	T3
**FTY**	8	12	12	16	10	13	17	17
**DMF**	13	15	12	22	15	18	13	22
**NAT**	15	12	18	20	15	11	18	20
**UNTR**	7	9	3	12	7	10	3	12
**CT**	7	25	7	28	7	25	7	29
Cytokines	Basal sample size				Stimulated sample size			
	T0	T1	T2	T3	T0	T1	T2	T3
**FTY**	na	na	na	11	na	na	na	13
**DMF**	na	na	na	21	na	na	na	22
**NAT**	na	na	na	18	na	na	na	19
**UNTR**	na	na	na	10	na	na	na	9
**CT**	na	na	na	24	na	na	na	24

The number of individuals evaluated at each time point (T0, T1, T2, T3) is detailed at the basal level and after stimulation. Age refers to T3. EDSS, Expanded Disability Status Scale; na, not applicable.

All subjects were monitored before vaccination (T0), 1 month after the second dose (T1), 6 months after the second dose (T2), and 1 month after the third dose (T3). Individuals diagnosed as anti-SARS-CoV-2 nucleocapsid antibody positive (using anti-SARS-CoV2-N kit, Roche) during the surveillance period were excluded from the study.

The study was approved by the local Ethical Review Board prot. N° 177/20021/EX2492/CE and prot. N° 336/2021/CE. The enrolled individuals signed written informed consent.

### Peripheral blood mononuclear cell isolation and activation

Peripheral blood mononuclear cells (PBMCs) were isolated using BD Vacutainer Mononuclear Cell Preparation Tubes (CPT) technology (BD Biosciences cat. 362780). CPT tubes, containing sodium heparin as an anticoagulant, liquid density medium, and an inert gel barrier, allowed the use of the same tube for primary sample processing. In detail, whole blood was drawn directly in the CPT tube and processed within two hours from collection. Immediately before centrifugation, CPT was gently inverted 8 to 10 times, and centrifuged at room temperature at 1800 rcf for 30 minutes. PBMC layer was aspirated and washed once with Phosphate Buffered Saline 1X (PBS 1X), centrifuged at 300 rcf for 15 minutes, and a second time with full RPMI 1640 medium (supplemented with 10% of Fetal bovine Serum-FBS, Sodium Pyruvate and Glutamine), then centrifuged at 300 rcf for 10 minutes. PBMCs resuspended in full RPMI were diluted with an equal volume of freezing media consisting of FBS with 20% dimethyl sulfoxide (DMSO, final concentration of 10%). Samples were stored at -80°C using a Mr Frosty container to achieve the optimal cell rate of cooling close to -1°C/minute, then transferred at -150°freezer until their use.

To assess activation, 5x10^5^ PBMCs were incubated in 100 µl of complete media with 1µg/mL of anti-CD28 (Invitrogen cat. 16–0289-85) and with or without 100 ng of Peptivator SARS-CoV-2 Prot-S (Miltenyi 130–126-701) for 23 hours at 37°C with 5% CO2. We performed the stimulations as physiologically as possible, e.g. avoiding membrane permeability blockers, which are widely used in research but alter normal cellular mechanisms (11, 25). This allowed us to assess physiological cytokine release both basally and after stimulation, and to assess markers of activation that are physiologically present at the cell surface rather than intracellularly after artificial accumulation.

After incubation, supernatants were harvested and stored at -80°C for subsequent cytokine analysis. Cells were washed with PBS 1X and stained for flow cytometry measurements.

### Flow cytometry measurements

We profiled 57 T-cell traits and 23 B-cell traits by 2 multicolor cell panels described below.

#### B-T panel

Basal and stimulated PBMC samples were stained with 15 antibodies (listed in **Supplementary Table 1**) for B and T cell characterization for 30 minutes at +4°C in the dark. After incubation, samples were washed with PBS 1X and analyzed by FACS ARIA cytometer (BD Biosciences). The gating strategy is described in **Supplementary Figure 1**.

#### Spike B panel

To characterize spike-specific B cells, 1x10^6^ thawed PBMCs were incubated with spike tetramer obtained by mixing the biotinylated SARS-CoV-2 spike protein (R&D BT10549) with the allophycocyanin (APC)-conjugate streptavidin (R&D cat. F0050) for 1 hour at +4°C using 4:1 (spike:streptavidin) molar ratio (11). PBMCs were then washed with Stain Buffer BSA (BD cat. 554657) and incubated with six further markers, listed in **Supplementary Table 1**, for 30 minutes at +4°C. After staining, cells were washed with PBS 1X and analyzed with a FACS ARIA cytometer. The gating strategy of the spike B cell panel is described in **Supplementary Figure 2**.

Cell types were evaluated as the frequency with respect to hierarchically higher cell populations and as count corresponding to the number of cells divided by the maximum time of cell acquisition. Flow cytometric data were manually gated by using FACS DIVA software (version 8.0.1).

### Detection of SARS-CoV-2 antibodies

Blood samples were collected in a vacutest tube with gel and clot activator. Sera were isolated within two hours after blood sampling and stored at -80°C until use. Detection of anti‐spike and anti-nucleocapsid antibodies was performed by electrochemiluminescence immunoassays using Elecsys® Anti-SARS-CoV2-S and Elecsys® Anti-SARS-CoV2-N kits (Roche), respectively, and the automated Cobas e-411 analyzer (Roche), according to manufacturer’s instructions. Anti-S results are expressed as units per ml (U/ml).

### Cytokine measurement

Cytokine production was assessed on cell supernatants from patients and controls at T3, at the baseline, and after stimulation, by using Luminex multiplex technology and the Bio plex 200 System Reader (BioRad). The human cytokine magnetic 10-plex panel kit (Invitrogen LHC0001M) was used to quantify human granulocyte-macrophage colony-stimulating factor (GM-CSF), interferon (IFN)-gamma, interleukin (IL)1-beta, IL2, IL4, IL5, IL6, IL8, IL10 and Tumor Necrosis Factor (TNF)-alpha, according to manufacturer’s instructions. Each sample was measured in duplicate. For each sample, the fluorescence intensity of each cytokine was normalized by the number of live lymphocytes measured in the same well.

### Statistical analysis

The normal distribution of the measured traits was assessed using the Kolmogorov–Smirnov test. Wilcoxon-Mann-Whitney test was applied to evaluate statistically significant differences between patients treated with DMTs and healthy controls or untreated patients. The analysis was performed by R software v.4.3.2. The nominal P-value of 0.05 was adjusted for multiple tests based on the number of traits and conditions simultaneously assessed.

In more detail,

- The immune characterization evaluated 66 immune cell traits (comprising 29 cell counts and 37 cell frequencies, see **Supplementary Table 2**) across 7 comparisons between groups (i.e. FTYvsCT, DMFvsCT, NATvsCT, UNTRvsCT, FTYvsUNTR, DMFvsUNTR, and NATvsUNTR, all at basal level). Thus, the nominal P-value of 0.05 was divided by the 66 immune traits and by 7 comparisons simultaneously assessed, resulting in a significance threshold of 1.08x10^-4^.
- Spike B cells, expressed as count and relative frequency, were evaluated at 3 time points after vaccination (T1, T2, and T3) and compared to T0 within each group of MS patients (FTY, DMF, NAT, UNTR) or healthy controls (CT). Thus, the nominal P-value of 0.05 was divided by 2 immune traits (spike B cells count and frequency) and by 3 comparisons (T0vsT1, T0vsT2, and T0vsT3, see **Supplementary Table 3**), resulting in a significance threshold of 8.33x10^-3^.
- Spike B cells of each MS group (FTY, DMT, NAT, or UNTR) were also compared to healthy controls (CT). In this case, the nominal P-value of 0.05 was divided by 2 immune traits simultaneously measured (Spike B cell count and frequency), 7 comparisons considered (i.e. FTYvsCT, DMFvsCT, NATvsCT, UNTRvsCT, FTYvsUNTR, DMFvsUNTR, and NATvsUNTR), and three time points (T1, T2, and T3, see **Supplementary Table 4**), resulting in a significance threshold of 1.19x10^-3^.
- Anti-S antibodies were compared between each MS group (FTY, DMF, NAT, UNTR) and healthy controls, thus the nominal P-value of 0.05 was divided by 4 MS groups, resulting in a significance threshold of 1.25x10^-2^ (see **Supplementary Table 5**).
- Comparing cell activation among groups, we considered 12 activated cell types and 14 comparisons (i.e. FTYvsCT, DMFvsCT, NATvsCT, UNTRvsCT, FTYvsUNTR, DMFvsUNTR, and NATvsUNTR, both at basal level and after stimulation, see **Supplementary Tables 6, 7**, respectively) simultaneously. Thus, the nominal P-value of 0.05 was divided by 12 immune traits and 14 comparisons, resulting in a significance threshold of 2.98x10^-4^.
- Comparing cell activation at basal vs stimulated levels within each group (FTY, DMF, NAT, UNTR, CT), the nominal P-value of 0.05 was divided by 12 activated cell types simultaneously assessed, thus the significance threshold was 4.16x10^-3^ (see **Supplementary Table 8**).
- Ten cytokine levels were compared between each MS group (FTY, DMT, NAT, UNTR) and healthy controls. Thus, the P-value of 0.05 was divided by 10 cytokines and by 14 comparisons simultaneously evaluated (i.e FTYvsCT, DMFvsCT, NATvsCT, UNTRvsCT, FTYvsUNTR, DMFvsUNTR, and NATvsUNTR, both at basal level and after activation), resulting in a significance threshold was 3.57x10^-4^ (see **Supplementary Tables 9, 10**).
- Comparing basal vs stimulated cytokine production within each group (FTY, DMF, NAT, UNTR, CT), the P-value of 0.05 was divided by the 10 cytokines simultaneously assessed, resulting in a significance threshold of 5.0x10^-3^ (see **Supplementary Table 11**).

**Supplementary Table 12** summarizes the significance thresholds applied in each experiment.

The correlation between spike B cells and anti-S antibodies was assessed by Spearman correlation.

We matched MS patients with controls having an average similar age and male/female ratio to avoid any bias due to these variables. We did not use EDSS and disease duration as covariates due to the small sample size of each group (FTY, DMF, NAT, UNTR). Furthermore, EDSS and disease duration were on average quite similar among DMT groups and untreated patients.

## Results

### Immune characterization of MS patients under three DMTs

**Figure 1:**
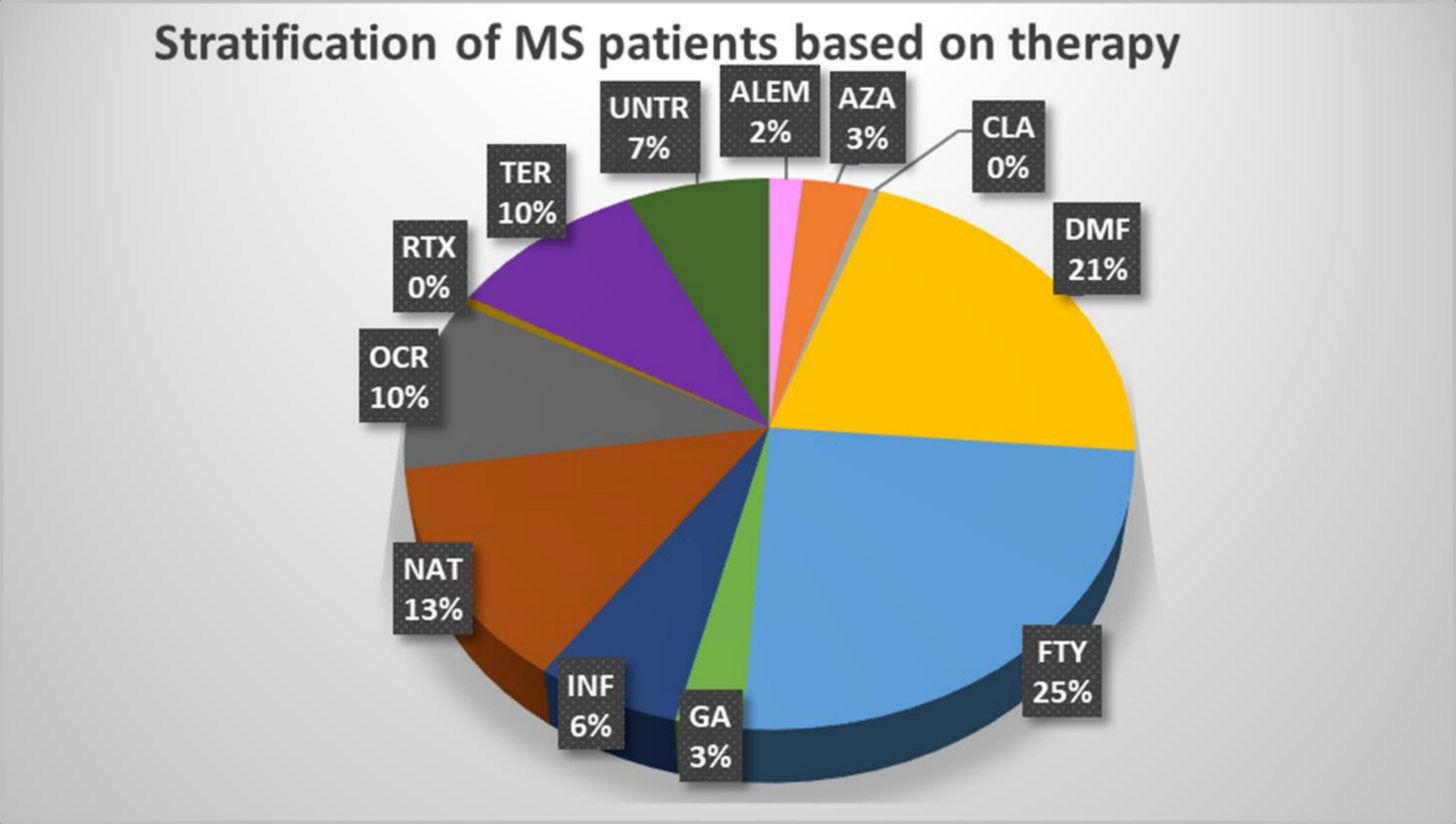
Stratification of MS patients based on therapy. The pie chart shows the percentage of each treatment used in the MS patients who participated in the study. ALEM, alemtuzumab (n.3); AZA, azathioprine (n.6); CLA, cladribine (n.1); DMF, dimethyl fumarate (n.39); FTY, fingolimod (n.46); GA, glatiramer acetate (n.5); IFN, interferon (n.11); NAT, natalizumab (n.24); OCR, ocrelizumab (n.19); RTX, rituximab (n.1); TER, teriflunomide (n.18); UNT, untreated (n.13).

We profiled 66 immune cell traits, including 17 B cell and 49 T cell phenotypes, in 79 MS patients stratified by treatment and 32 healthy subjects at T0, T1, T2 and T3. **Supplementary Table 2** and **Supplementary Figures 1, 2** provide a detailed description of the immune traits that were characterized and how each group of treated patients compared with untreated patients and healthy subjects. In addition, **Figure 1** shows the number of available patients and the basis for the focus on FTY, DMF, and NAT as the treatments most often employed with those patients. **Table 1** describes the main features of each selected group and the number of samples evaluated for each measurement.

We initially compared immune status in patients treated with DMTs vs healthy controls at T3 (the time point with the largest sample size). Significant differences were seen in immune trait levels.

**Figure 2:**
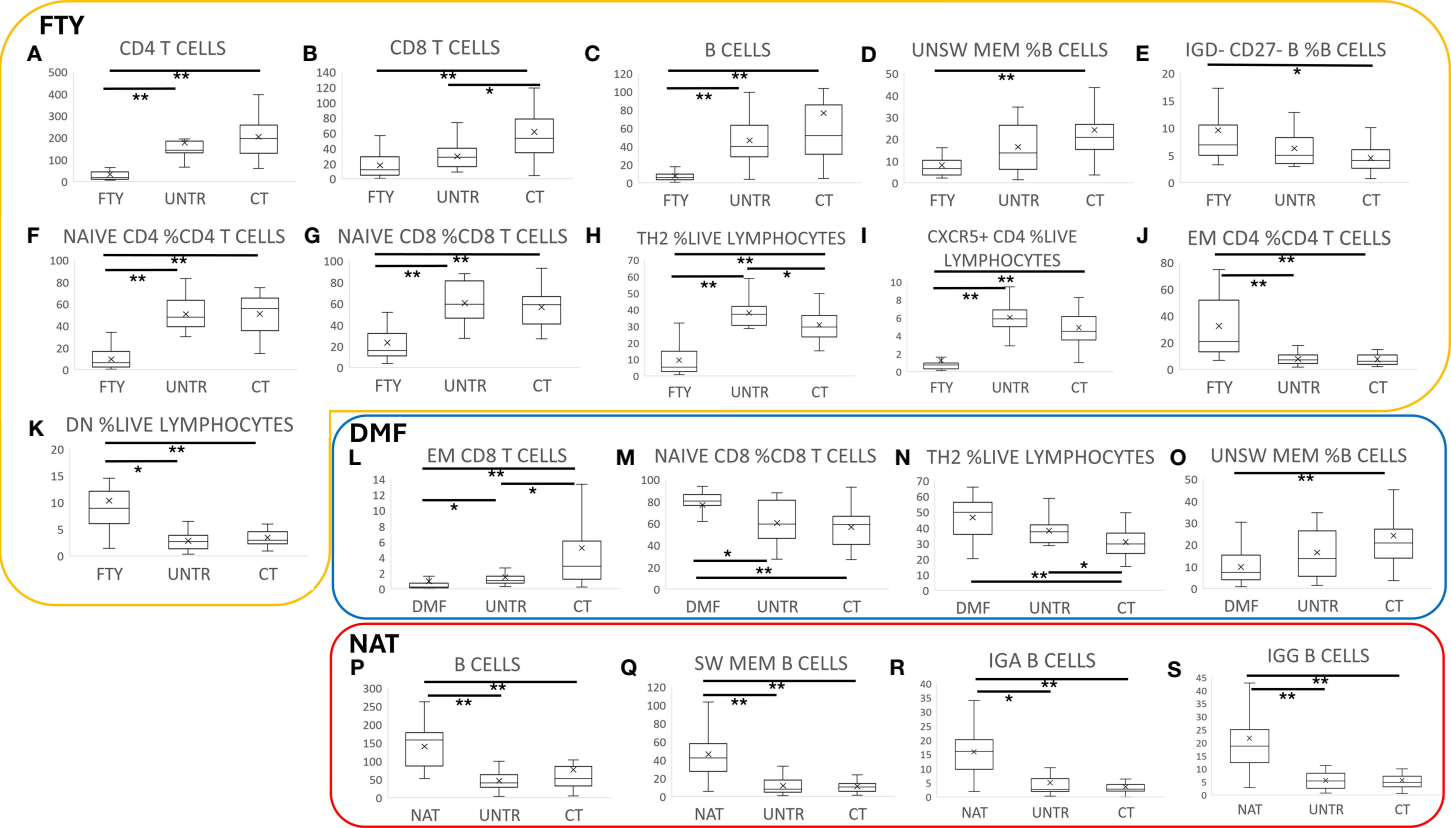
Immune characterization of MS patients stratified by therapy. Comparison of MS patients stratified by therapy to untreated patients and controls. Each boxplot graph represents a specific cell type expressed as cell count (cells/time, graphs **A-C, L, P-S**) or as cell frequency with respect to a hierarchically higher cell population (graphs **D-K**, **M-O**). The “x” within each box represents the average value, whereas the horizontal bar in each box indicates the median value. Graphs within the yellow box refer to FTY treatment, the blue box to DMF, and the red one to NAT- treated patients. P-values ranging from 0.05 to 1.08x10^-4^ are indicated with “*” and considered nominally significant; P-values below 1.08x10^-4^ are indicated with “**” and considered significant. See Methods for specifications about multiple test corrections.

FTY therapy correlated with a significant reduction of all lymphoid cell subset counts, such as CD4, CD8, and B lymphocytes (**Figures 2A–C**, P=1.8×10^-09^, P=1.2×10^-05^, P=5.4×10^-12^), accompanied by decreased unswitched memory and increased IgD- CD27- and naïve B cell frequencies (**Figures 2D, E**, P=1.8×10^-07^, P=1.2×10^-04^, P=2.2×10^-03^).

FTY treatment also led to an alteration of T cell frequencies. Indeed, the relative counts of Naïve CD4 and CD8, Th2, and CXCR5+ helper T cells strongly decreased (**Figures 2F–I**, P=1.3×10^-09^, P=4.9×10^-06^, P=1.3×10^-07,^P=2.4×10^-07^), whereas effector memory (EM) CD4 and CD4-CD8- (DN) T cells and Th1 increased (**Figures 2J, K**, P=1.3×10^-06^, P=3.7×10^-05^, P=0.02).

DMF therapy correlated with a general reduction of absolute cell counts, particularly evident in the memory compartment of CD8 cells with a consequent increase of Naïve CD8 cell frequency (**Figures 2L, M**, P=1.1×10^-06^, P=8.9×10^-05^). We also observed an increase of Th2 and a reduction of unswitched memory B cell frequencies (**Figures 2N, O**, P=6.7×10^-05^, P=2.0×10^-05^). These results are in line with previous studies (26, 27).

NAT treatment was associated with an increase in B cells (**Figure 2P**, P=2.0×10^-05^) that was particularly evident in the memory subsets, especially in switched memory, IgA+, and IgG+ B cells (**Figures 2Q–S**, P=2.7×10^-09^, P=3.9×10^-09^, P=3.6×10^-08^), which was consistent with previous studies (28–30).

Untreated samples were characterized by a nominal reduction of CD8+ T cells (**Figure 2B**, P=9.3×10^-03^), especially the effector memory subset (**Figure 2L**, P=5.7×10^-03^), with a consequent increase of CD4 T cell frequency (P=4.2×10^-03^).

Notably, the described differences in immune traits between treated patients and healthy controls were also observed between treated and untreated patients, albeit at a lower level of significance. This, and the fact that the findings detected at T3 were consistent across the other three time points, suggests that they are indeed mediated by the DMTs (see **Supplementary Table 2**).

### SARS-CoV-2 specific B cell response after vaccination

To characterize the immune response specifically due to SARS-CoV-2 vaccination, we evaluated the level of SARS-CoV-2 anti-spike B cells (expressed as counts and relative frequencies) in MS patients and controls at the four time points.

**Figure 3:**
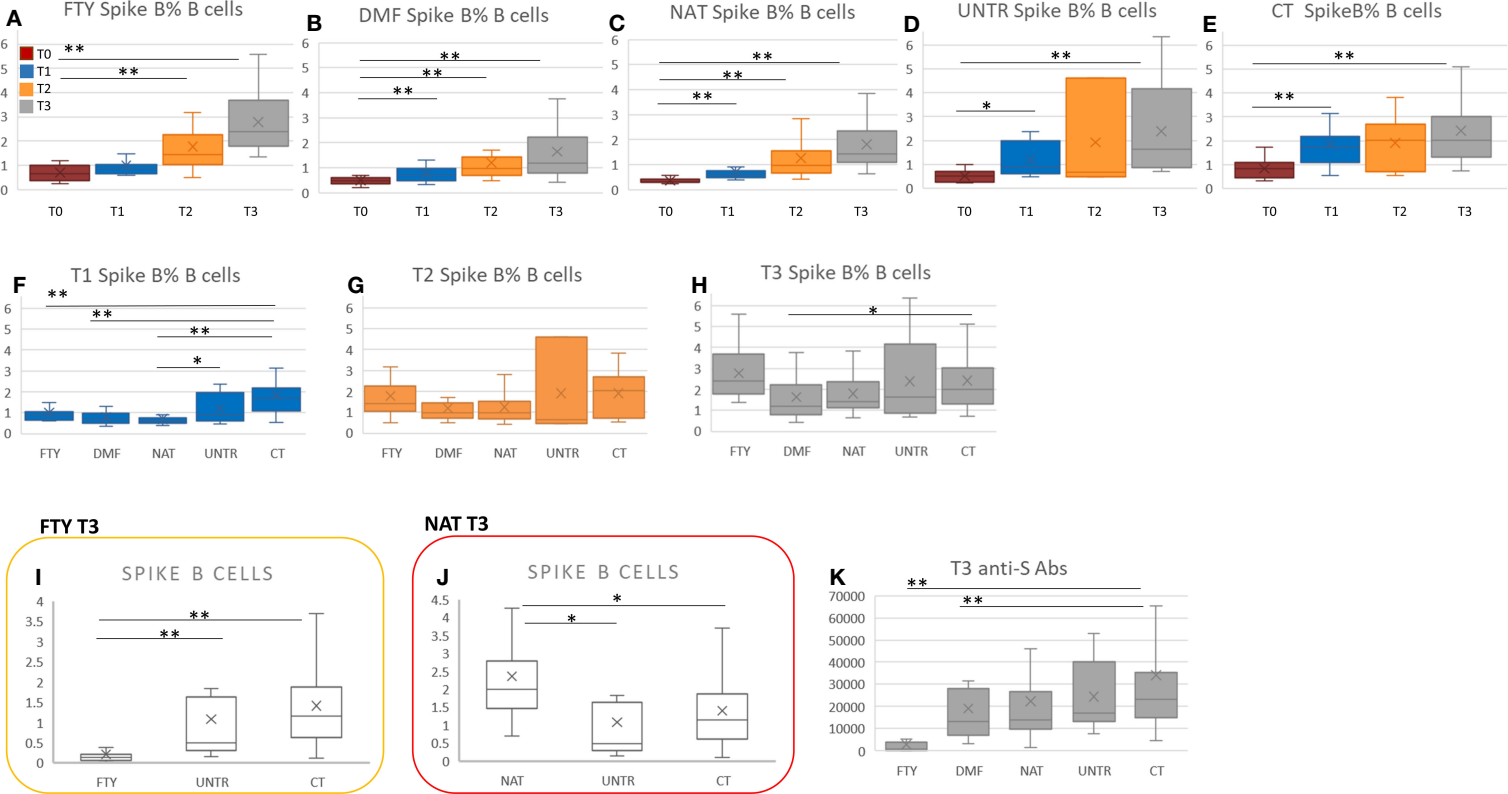
Spike-specific B cells in MS patients vs controls at each time point. **(A-E)** Comparison of spike-specific B cell frequency at the four time points (from T0 to T3) in each MS group and healthy controls. P-values ranging from 0.05 to 8.33x10^-3^ are indicated with “*” and considered nominally significant; P-values below 8.33x10^-3^ are indicated with “**” and considered significant. **(F-H)** Comparison of spike-specific B cell frequency among the MS groups and healthy controls considering T1, T2, and T3 separately. **(I)** Comparison of spike B cell count among FTY-treated, untreated patients and controls at T3. **(J)** Comparison of spike B cell count among NAT-treated, untreated patients and controls at T3. P-values ranging from 0.05 to 1.19x10^-3^ are indicated with “*” and considered nominally significant; P-values below 1.19x10^-3^ are indicated with “**” and considered significant. **(K)** Comparison of anti-S antibodies between each MS group and controls at T3. P-values ranging from 0.05 to 1.25x10^-2^ are indicated with “*” and considered nominally significant; P-values below 1.25x10^-2^ are indicated with “**” and considered significant. See Methods for specifications about multiple test corrections.

In each group, we observed a general increase of spike-specific B cell frequency moving from T1 to T2 to T3 (**Figures 3A–E**; **Supplementary Table 3**).

In particular, comparing MS patients with controls at T1, the percentage of spike-specific B cells was significantly lower in all categories of MS patients than in controls. The finding was significant in FTY, DMF, and NAT-treated patients (P<2.8×10^-04^), but the same trend was also present when comparing treated with untreated patients (**Figure 3F**; **Supplementary Table 4**).

At T2 and T3, we observed no significant difference in spike-specific B cell frequency between MS patients treated with FTY, DMF, and NAT and controls (**Figures 3G, H**). Nevertheless, because FTY-treated patients were characterized by lymphopenia (**Figures 2A–C**), also their spike-specific B cell level was strongly reduced (**Figure 3I**, P=1.3×10^-09^).

In NAT, the increase of the B cell compartment, described in the previous paragraph, also led to an increment in anti-spike B cell level (**Figure 3J**, P=0.008), which, however, did not significantly rise as relative frequency (**Figures 3G, H**).

No significant differences in anti-spike B levels were observed between untreated patients and controls at T2 and T3 (**Figure 3H**).

Overall, moving from T1 to T3, the difference in anti-spike B cell frequency between MS patients and controls was no longer significant, supporting the importance of vaccine boosters in MS patients (see Discussion).

### Correlation between SARS-CoV-2 specific B cells and anti-spike antibodies

The level and percentage of anti-spike specific B cells were then correlated with the levels of anti-spike antibodies previously evaluated in a larger cohort (4) that included a subset of patients assessed here for the cellular response. At T3, FTY- and DMF-treated patients showed a significant reduction of anti-spike antibodies compared to healthy subjects (**Figure 3K**).

**Figure 4:**
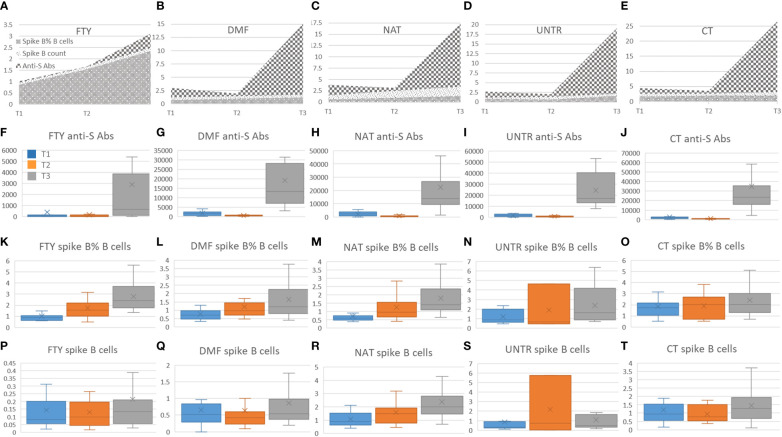
Correlation among spike B cells and anti-S antibodies. **(A-E)** Stacked area graphs represent comparisons of anti-S and spike B cell count and frequency levels in each category. To use a unique scale, anti-S measurements (U/ml) have been divided by 1000. Legend for stacked area graphs is in **(A)**. **(F-T)** Each boxplot represents the anti-S Abs expressed in U/ml (second row, **F-J**) and the corresponding spike B cells frequency (third row, **K-O**) and count expressed in number of cell/time (fourth row, **P-T**). Boxplots are color-coded as described in **(F)**.

Focusing on each category (FTY, DMF, NAT, UNTR, CT), we observed no significant correlation between anti-spike B cells and anti-spike antibodies (**Figures 4A–E**); rather we saw a decrease of anti-spike antibodies in all categories at T2 than T1 (**Figures 4F–J**), unaccompanied by a reduction of spike-B cell frequency (**Figures 4K–O**) and level (**Figures 4P–T**). Indeed, as shown in **Figure 4**, the frequency of spike B cells increased from T1 to T3. This suggests that, although the antibody titer declines six months after vaccination, spike-specific B cells are nevertheless present and ready to respond with the production of antibodies in the event of a new SARS-CoV-2 infection.

### B and T cell response following stimulation with Peptivator

To evaluate the activation status of immune cells both at basal level and after stimulation, we exposed lympho-monocytes from MS patients and controls to a pool of SARS-CoV-2 spike peptides (Peptivator) and assessed four activation cell surface markers (CD69, HLA-DR, CD25, and CD137) in helper (CD4+), cytotoxic (CD8+) and B (CD19+) lymphocytes.

In every group (FTY, DMF, NAT, UNTR, CT), no significant change in the activation status was seen from T0 to T3; we reported results obtained at T3 as the time point with the largest sample size (see **Supplementary Tables 6–8**). [Results of comparing patients treated with different DMTs with healthy controls or untreated patients at the other time points are reported in **Supplementary Tables 6–8**].

**Figure 5:**
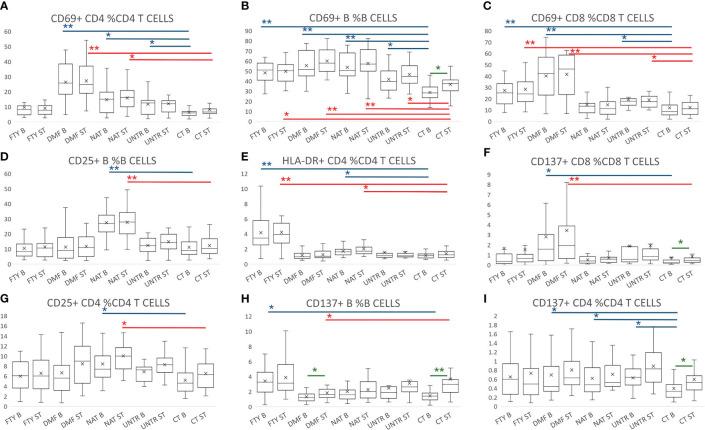
Lymphocyte activation following stimulation with Peptivator. **(A–I)** Boxplot graphs representing activated cell frequencies in MS patients stratified by therapy, untreated patients, and controls. Each group is represented at the basal level and following stimulation with Peptivator. Comparing basal or stimulated samples among groups, P-values ranging from 0.05 to 2.98x10^-4^ are indicated with “*” and considered nominally significant; P-values below 2.98x10^-4^ are indicated with “**” and considered significant. Comparing basal vs stimulated samples in the same group, P-values ranging from 0.05 to 4.16x10^-3^ are indicated with “*” and considered nominally significant; P-values below 4.16x10^-3^ are indicated with “**” and considered significant. See Methods for specifications about multiple test corrections. For simplicity, only asterisks related to comparisons between MS groups and controls are reported, whereas they are omitted for treated vs untreated MS patient comparisons. Asterisks are color-coded: in blue refer to basal level comparison, in red to comparisons of stimulated samples, and in green to basal vs stimulated comparisons in the same group.

At the basal level, MS B and T cells were more activated compared to controls, and this was particularly evident for the CD69 activation marker (**Figures 5A–C**, top P=4.7×10^-09^). The other activation markers were differentially upregulated in MS patients depending on the treatment used. For instance, the upregulation of CD25+ B cell frequency was statistically significant only in NAT (P=1.2×10^-07^, **Figure 5D**). Similarly, the frequency of HLA-DR+ CD4 cells was upregulated in FTY- and NAT-treated patients (**Figure 5E**, P=1.5×10^-06^, and P=5.8×10^-04^, respectively), whereas CD137+ CD8 lymphocytes increased in DMF compared to CT (**Figure 5F**, P=9.7×10^-04^). These data indicate a general upregulation of activated B and T cells at the basal level in MS patients, with an additional effect on activation due to the specific treatment used (**Supplementary Table 6**).

Similarly, comparing Peptivator-stimulated samples, we detected more activated cells in MS patients (in all treatments as well as untreated samples) with respect to controls. In more detail, the frequency of CD69+ B and CD69+ CD8, and HLA-DR+ CD4 T cells increased in FTY (**Figures 5B, C, E**, P=5.3×10^-04^, P=1.9×10^-05^, P=1.7×10^-06^). Higher activation was also observed in DMF, where it was particularly striking in CD69+ lymphocytes (**Figures 5A–C**: CD4+ P=1.5×10^-09^; B cells, P=3.2×10^-09^; CD8+, P=1.0×10^-07^), but was also significant in other CD8 T subsets (e.g. CD137+ CD8 T cells, **Figure 5F**, P =2.9×10^-04^). In NAT the strongest activation was observed in B cells (CD25+ and CD69+ B cells, **Figures 5B, D**, P=5.2×10^-07^ and P=1.5×10^-05^, respectively), and to a lesser extent in CD4+ T cells (**Figure 5E**, CD69+ CD4, P=5.2×10^-04^; CD25+ CD4, P=8.5×10^-04^). In untreated patients, the increased activation was nominally significant in CD69+ CD8 T and B cells (**Figure 5A**; **Supplementary Table 7**).

Importantly, comparing stimulated samples to their corresponding basal samples, we observed that the T and B cell responses (CD137+ B, CD137+ CD4, and CD69+ B cells) were consistently more pronounced and significant in controls than in MS patients (**Figures 5B, G–I**; **Supplementary Table 8**). Overall, these data indicate that MS lymphocytes are more activated (and likely non-specifically aggressive) at the basal level, but less responsive to specific stimuli. Again, there is thus an indicated need for booster to augment a specific immune response.

### Cytokine production following stimulation with Peptivator

To further analyze the immune response following stimulation in MS patients and controls, we also measured the level of ten cytokines from supernatants of lympho-monocyte cultures at baseline and after Peptivator stimulation at T3.

**Figure 6:**
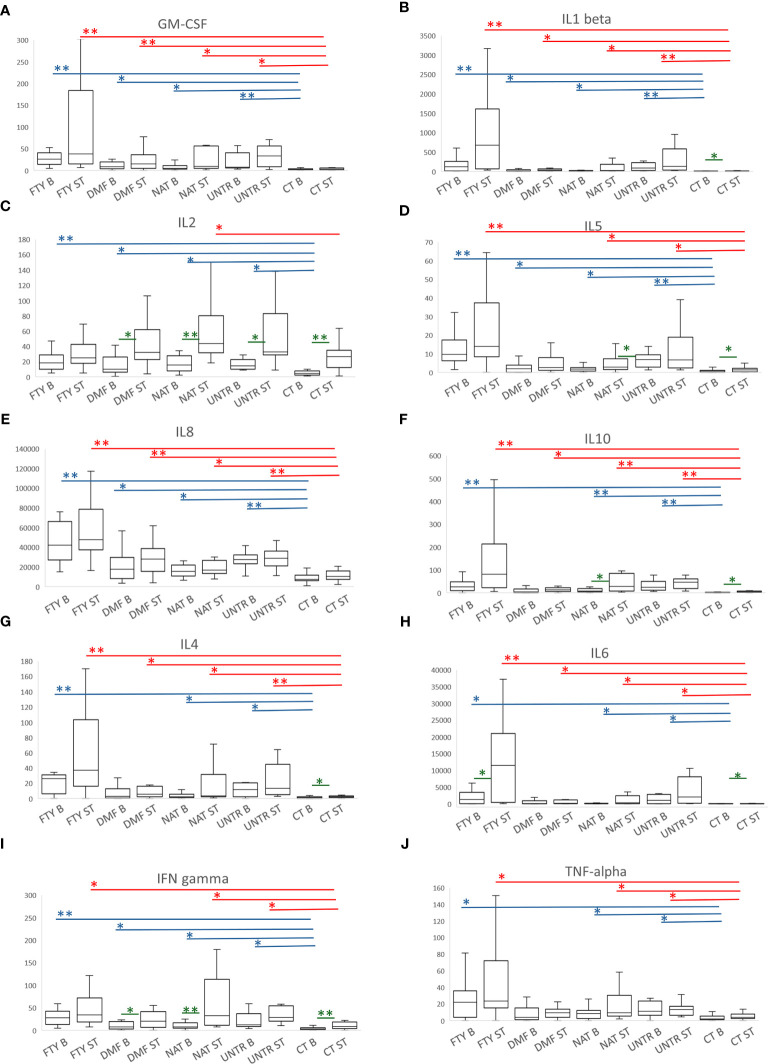
Cytokine production following stimulation with Peptivator. **(A–J)** For each cytokine measured, boxplots of MS patients treated with FTY, DMF, NAT, or UNTR and healthy subjects are shown. Each boxplot graph represents a specific cytokine expressed as fluorescence intensity/cell (see Methods). Comparing basal or stimulated samples among groups, P-values ranging from 0.05 to 3.57x10^-4^ are indicated with “*” and considered nominally significant; P-values below 3.57x10^-4^ are indicated with “**” and considered significant. Asterisks are in blue for comparison at the basal level, and in red for comparison of stimulated samples. Comparing basal vs stimulated samples in the same group, P-values (colored in green) ranging from 0.05 to 0.005 are indicated with “*” and considered nominally significant; P-values below 0.005 are indicated with “**” and considered significant. As for **Figure 4**, only asterisks related to comparisons among MS groups and controls are reported.

At the basal level, we observed higher cytokine production in all MS groups compared to controls at T3 (**Figure 6**). The increased production was observed for both pro-inflammatory proteins, such as GM-CSF, IL1-beta, IL2, IL5, and IL8 (**Figures 6A–E**), and anti-inflammatory proteins, such as IL10 (**Figure 6F**), indicating that the higher cytokine secretion in MS patients was likely independent of cytokine function (**Supplementary Table 9**).

Likewise, after Peptivator stimulation, we observed a generally higher cytokine production in all MS patients compared to healthy controls, particularly evident in FTY individuals, in whom 7 (GM-CSF, IL1-beta, IL5, IL8, IL10, IL4, IL6) of 10 cytokines were significantly upregulated (**Figures 6A, B, D–H**, top P=6.7×10^-09^), whereas IFN-gamma and TNF-alpha were only nominally increased (**Figures 6I, J**; **Supplementary Table 10**).

Importantly, when we compared stimulated samples to the corresponding basal samples, we observed the strongest upregulation of cytokine production in CT samples and the lowest upregulation in FTY patients (**Supplementary Table 11**).

Overall, these data are in line with those observed for activation cell markers, confirming that, despite an increased basal immune activation, immune cells from MS patients are consistently less responsive to a specific stimulus.

## Discussion

**Figure 7:**
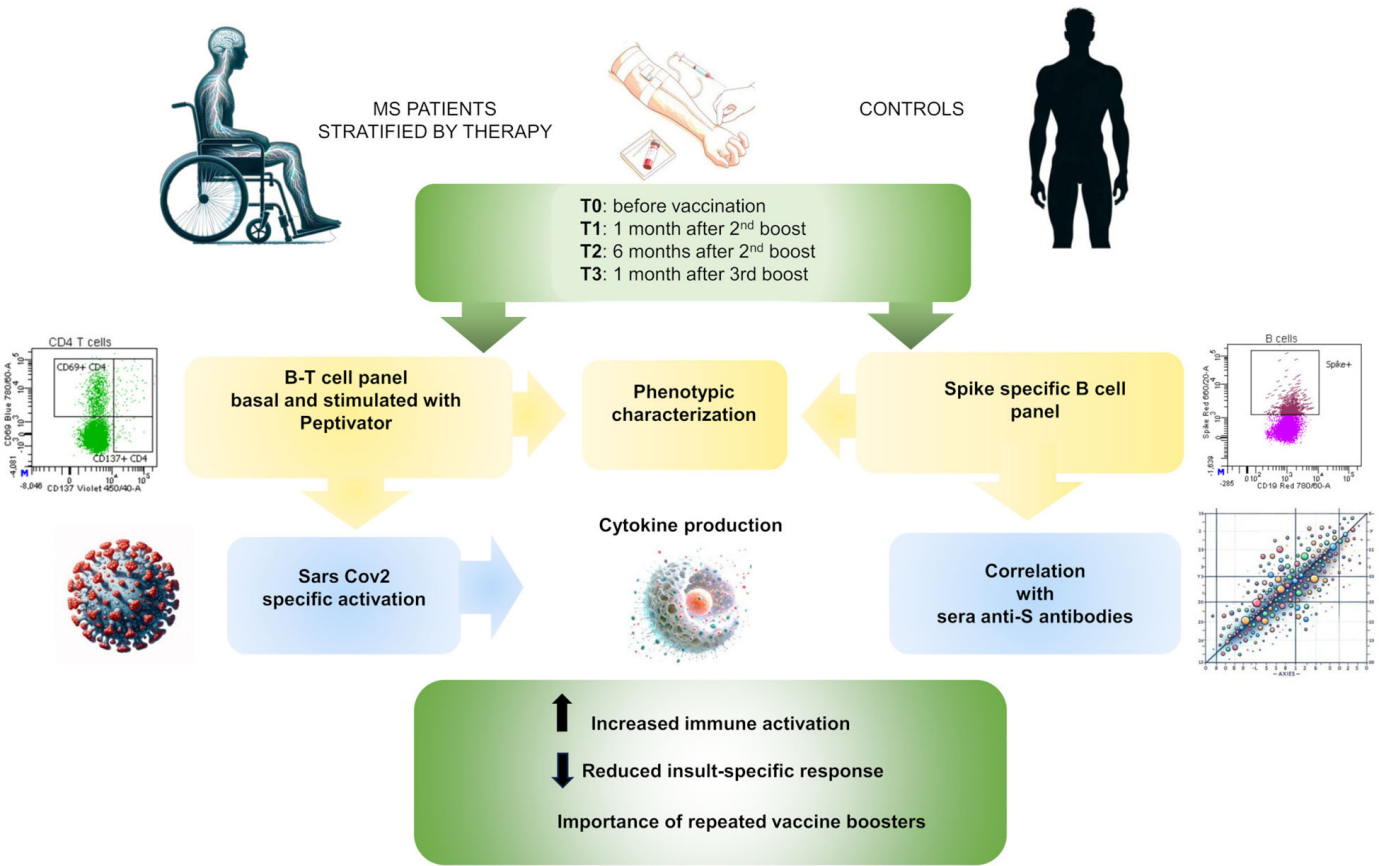
Study overview. Schematic workflow of the present study.

We reported a broad evaluation of B and T immune cell traits in MS patients under treatment with three commonly used therapies- FTY, DMF, and NAT- and UNTR patients compared to healthy subjects. These immune features were assessed both at the basal level and in response to immune stimuli, vaccination against SARS-CoV-2 virus *in vivo* and activation with a pool of spike protein peptides in cultured cells. An overview of the study workflow is shown in **Figure 7**.

In line with previous studies (10, 31–33), we observed pronounced immune cell dysregulation in FTY-treated patients, characterized by reduction of all lymphocyte cell types considered. However, in contrast to a previous study (34), we also detected a significant reduction in Th2 frequency accompanied by a nominal increase in Th1. This drug, modulating sphingosine receptors, is known to trap naïve and central memory T cells in secondary lymphoid organs, thus reducing the T naïve cell compartment, as we observed. Notably, all lymphocyte subsets evaluated in this study were reduced in FTY-treated patients, and the increase in a specific cell frequency is thus only due to the more pronounced reduction in one cell type with respect to another. For instance, naïve B cells decrease in FTY-treated patients and the previously observed increase in naïve B cell frequency (35) is a secondary effect due to the stronger decrease in memory (switched and unswitched) B cells. Similarly, effector memory T cells decline with FTY treatment, and their previously reported increase in frequency (34, 36), is primarily driven by the more pronounced reduction in naïve T cell level compared to effector memory T cells.

Consistent with previous studies, DMF- and NAT-treated patients showed lesser and more specific immune cell changes than those treated with FTY (26, 28, 31). These changes mainly affected cytotoxic memory cells in DMF-treated patients and memory B cells in NAT-treated patients. These differences were observed in DMT patients compared to both healthy and, to a lesser extent, untreated patients, suggesting that these effects are likely due to the pharmacological treatment rather than the pathology itself. Furthermore, the reduction in CD8+ T cells we observed in untreated patients is in line with their lower CD8 response, described in a previous study (37). The reduction in their level in peripheral blood could result from their displacement to the central nervous system, especially in MS lesions, where they are known to be the predominant T cell type (38). However, their pathogenic or regulatory involvement at the inflammatory site is still not clear.

When we assessed the immune system response *in vitro* using a pool of spike peptides, we found a basal activated state and a basal pro-inflammatory condition in MS patients compared to controls, accompanied by a reduced responsiveness to specific stimuli. This was seen both by evaluating cell surface activation markers and cytokine secretion, and was evident in all MS groups assessed, particularly in FTY-treated patients. These findings are in line with the premature immunosenescence observed in MS and in patients with other inflammatory conditions (10, 39, 40); it includes thymic involution (41), telomere length shortening (42), decline in the adaptive immune response, and an expansion of age-associated B cells (43), with a possible contribution to neurodegeneration (44–46). Overall, these changes also cause a concomitant increase in the pro-inflammatory state, a phenomenon known as inflammaging (47, 48), and a reduced immune response to vaccination (49).

As for the specific immune responses to the SARS-CoV-2 vaccine, we found that one month after the second booster dose (T1), all categories of patients had significantly lower frequency of anti-spike B cells than did controls. This difference was no longer significant one month after the third vaccine dose (T3), supporting the inference that booster vaccine is critical to achieving a specific anti-spike cell response comparable to that of healthy individuals in MS patients treated with these DMTs. However, spike B cell counts were significantly reduced in FTY-treated and, to a lesser extent, in DMF-treated patients, and increased in NAT-treated patients, further highlighting that important changes in the immune system may be caused by different DMTs. Furthermore, we observed no significant correlation between anti-spike B cells and anti-spike antibodies in patients under any of the three DMT regimens considered here, as well as in untreated patients and healthy controls (**Figures 4A–E**). Rather, we observed a decrease in anti-spike antibodies in all categories 6 months after the second vaccine dose (T2) compared to 1 month after that dose (T1), which, remarkably, was not accompanied by a decrease in the frequency and number of spike B cells. This suggests that even though circulating antibodies decrease at T2, spike B cells increase in frequency and are ready to respond when re-activated by an appropriate stimulus, and in particular, by additional doses of vaccine or natural infection.

Our work also has some limitations mainly related to the relatively small sample size and the inclusion of only 3 DMTs. Therefore, the observations reported here need to be extended using larger sample sizes, which may reveal further differences between patients and controls, and including additional DMTs, which may reveal some peculiarities of unassessed treatments.

Overall, this work can be considered as a representative example of the response of the immune system to vaccination in MS patients. The findings may apply to the extent and nature of the immune response of MS patients to other vaccines, including those against influenza, rubella, herpes zoster, and other pathogens. The results suggest an approach to appropriate recommendations for boosting strategies to achieve adequate immune responses. Furthermore, many of these considerations may apply to individuals with immunological impairment imposed by genetics, environmental factors, or treatment with immunosuppressive drugs.

## Data availability statement

The original contributions presented in the study are included in the article/**Supplementary Material**. Further inquiries can be directed to the corresponding author.

## Ethics statement

The study was approved by the local Ethical Review Board “Comitato Etico Regione Sardegna” prot. N° 177/20021/EX2492/CE and prot. N° 336/2021/CE. The studies were conducted in accordance with the local legislation and institutional requirements. The participants provided their written informed consent to participate in this study.

## Author contributions

VO: Conceptualization, Supervision, Writing – original draft, Formal analysis. VS: Writing – original draft, Investigation, Formal analysis. MMar: Formal analysis, Software, Writing – review & editing. SL: Investigation, Writing – review & editing. VL: Investigation, Writing – review & editing. MZ: Investigation, Writing – review & editing. MS: Formal analysis, Writing – review & editing. AL: Investigation, Writing – review & editing. ML: Investigation, Writing – review & editing. MGP: Investigation, Writing – review & editing. FV: Investigation, Writing – review & editing. GD: Investigation, Writing – review & editing. MGM: Investigation, Writing – review & editing. MMin: Investigation, Writing – review & editing. MFl: Writing – review & editing. MMas: Writing – review & editing. MPC: Resources, Writing – review & editing. RM: Investigation, Writing – review & editing. JF: Investigation, Writing – review & editing. LL: Investigation, Writing – review & editing. GF: Investigation, Writing – review & editing. MFr: Investigation, Writing – review & editing. DC: Investigation, Writing – review & editing. ECa: Investigation, Writing – review & editing. SP: Investigation, Writing – review & editing. PCh: Investigation, Writing – review & editing. MD: Writing – review & editing. PCa: Investigation, Writing – review & editing. PS: Writing – review & editing. RIZ: Investigation, Writing – review & editing. MLI: Conceptualization, Investigation, Writing – review & editing. MP: Conceptualization, Investigation, Writing – review & editing. ECo: Investigation, Writing – review & editing. EF Conceptualization, Writing – review & editing. FC: Conceptualization, Funding acquisition, Project administration, Writing – review & editing.
