# Supplementary material for: Implications of disease-modifying therapies for multiple sclerosis on immune cells and response to COVID-19 vaccination
Source: Front Immunol. 2024 Jul 15;15:1416464. doi: 10.3389/fimmu.2024.1416464 (PMC11284103; doi:10.3389/fimmu.2024.1416464)
Supplement: Supplementary file 1 [file DataSheet_1.pdf]

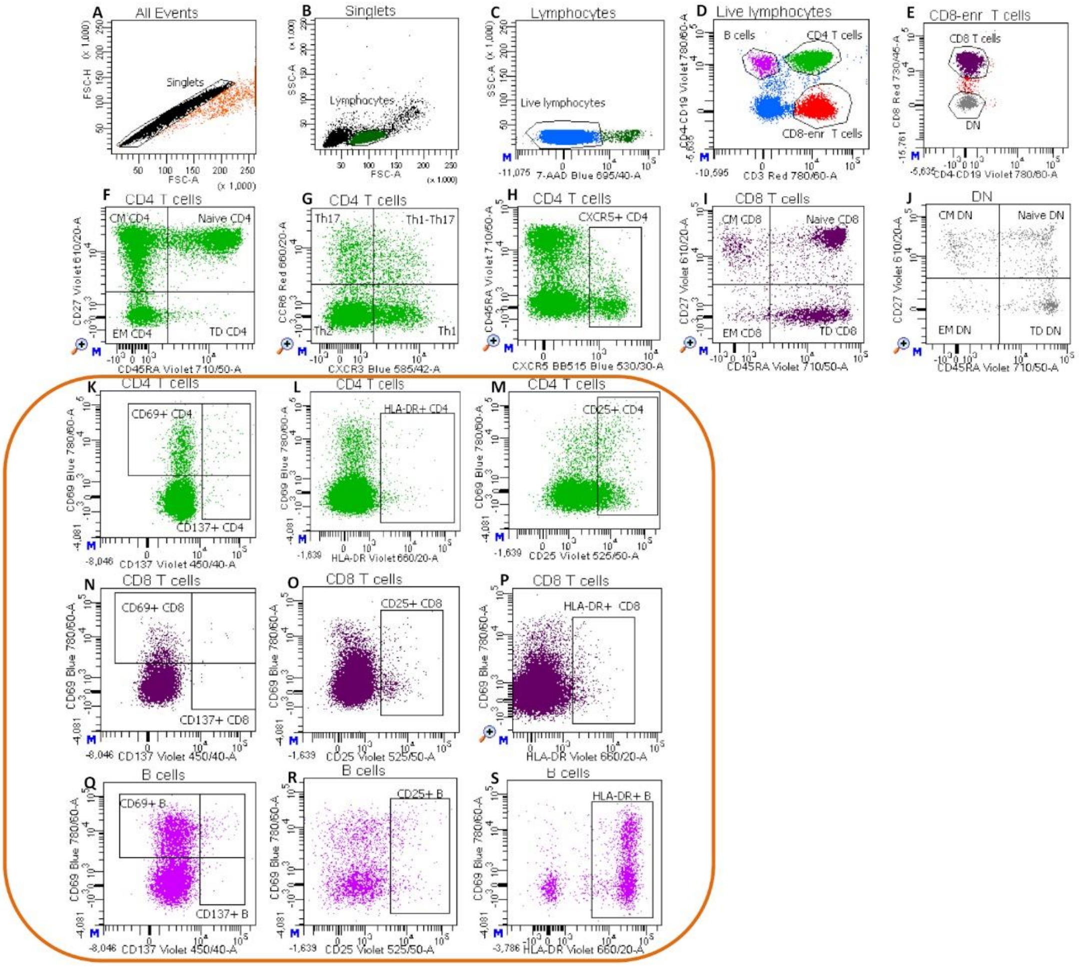

Supplementary Figure 1. Gating strategy for B-T panel. A) Singlets (black) and B) lymphocytes (dark green) were identified based on morphological parameters. C) Viable lymphocytes (blue) were negative for 7-amino actinomycin D (7-AAD) and D) divided into B cells (CD19+ CD3-, violet), CD4 T cells (CD4+ CD3+, green), and CD8-enr(iched) T cells (CD3+ CD4- CD19-, red). E) The latter were divided into CD8 T cells (CD8+, dark violet) and DN (CD4-CD8-, grey). CD4 T cells were divided into F) naïve, central memory (CM), effector memory (EM), and terminally differentiated (TD) based on CD45RA and CD27 expression and G) Th1, Th2, Th17, and Th1-Th17 based on CXCR3 and CCR6 expression. H) CXCR5+ CD4 T cells were also evaluated. I-J) The maturation stages of CD8 and DN T cells were also assessed, respectively. The activation markers CD69, CD137, HLA-DR, and CD25 were grouped in the orange quadrant and measured in CD4 T cells (K-M), CD8 T cells (N-P), and B cells (Q-R).

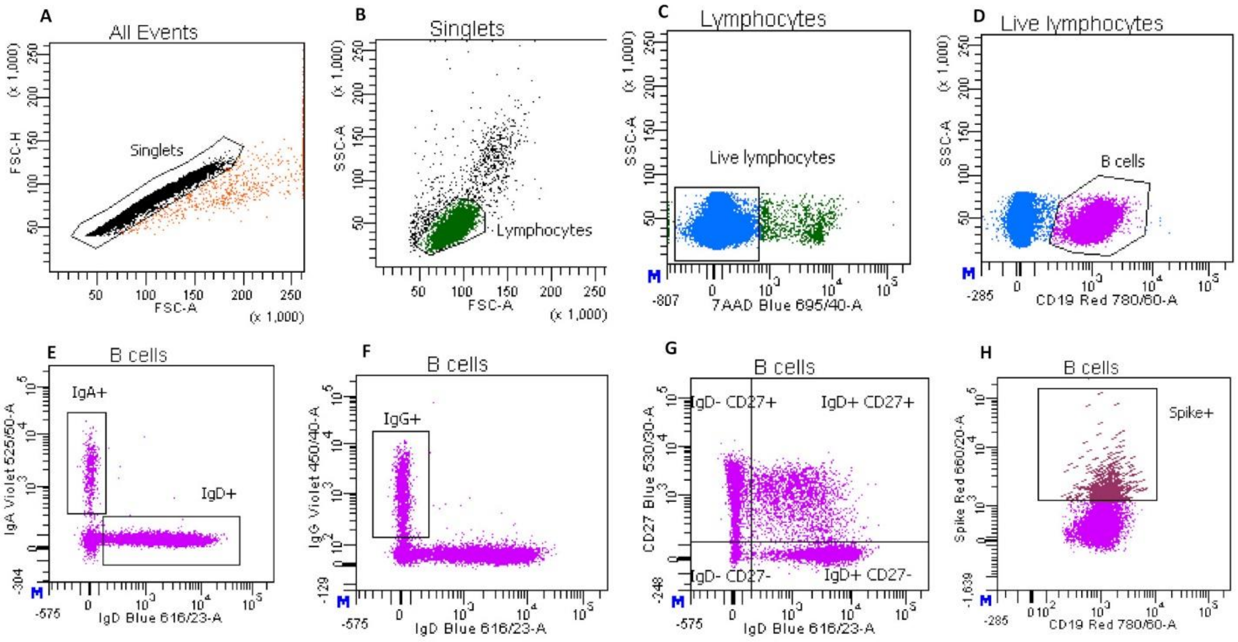

Supplementary Figure 2. Gating strategy for spike B cell panel. (A) Singlets (black) and (B) lymphocytes (dark green) were identified based on morphological parameters. (C) Viable lymphocytes (blue) were negative for 7-amino actinomycin D (7-AAD). (D) B cells (violet) were identified as CD19+ lymphocytes and divided into (E) IgA+, IgD+, (F) IgG+, (G) naïve (IgD+ CD27-), unswitched memory (IgD+ CD27+), switched memory (IgD- CD27+), and IgD- CD27-, and (H) spike+ B cells.
